# Medications for alcohol use disorder among patients with alcohol-associated cirrhosis: An underutilized intervention that could save lives

**DOI:** 10.1097/HC9.0000000000000202

**Published:** 2023-06-28

**Authors:** Sarah E. Wakeman

**Affiliations:** *Department of Medicine, Massachusetts General Hospital, Mass General Brigham, Office of the Chief Medical Officer Harvard Medical School, Boston, Massachusetts, USA*

In a recent issue of *Hepatology Communications*, Rabiee et al^1^ present findings from an evaluation of the impact of medications for alcohol use disorder (MAUD) on all-cause mortality among people with alcohol-associated cirrhosis. This study utilized a data set of veterans from 2008 to 2021, who had a diagnosis of alcohol use disorder (AUD), alcohol-associated cirrhosis (ARC), and a recorded AUDIT-C of 8 or higher in the year before their cirrhosis diagnosis. They then examined the impact of newly initiated acamprosate or oral naltrexone on all-cause mortality.

## MAIN FINDINGS

Among 9131 included individuals, fewer than 1 in 10 were treated with MAUD for a week or longer in the year after ARC diagnosis, which speaks to the tremendous treatment gap for AUD. MAUD treatment rates did increase over the study time period, from 4% in 2008 to 23% after 2017, which is a positive sign of increasing treatment access. Treatment duration was overall relatively short, with 61% treated for <3 months with MAUD. As with other types of substance use disorder (SUD) treatments, such as medication for opioid use disorder, disparities were seen in who was treated with MAUD, with the odds of MAUD in White patients 1.5 times higher than in Black patients. Hospitalization for AUD was associated with MAUD initiation, and an inpatient diagnosis code for AUD was the variable most strongly associated with receipt of MAUD. In contrast, those with decompensated cirrhosis were less likely to receive MAUD. Using propensity-matched cohorts, the authors found that, in the intention-to-treat analysis, MAUD treatment in the first year after ARC diagnosis was associated with a 20% reduced mortality. Duration of MAUD treatment was associated with a greater reduction in mortality. Treatment for >3 was associated with a 27% reduced hazard of dying.

## MAUD IS LIFESAVING

These findings demonstrate the lifesaving potential for MAUD among people with ARC and the urgent need to close the remaining treatment gap, particularly for those with liver disease. Amidst rising alcohol-associated mortality among younger individuals, the need for standardized approaches to ensure treatment for AUD has never been more urgent.^2,3^ The finding that only 1 in 10 individuals with a potentially fatal alcohol-associated health condition, who were accessing health care regularly and were known to have AUD, was treated speaks to the lack of integration of AUD treatment into general medical settings. Prior research has found that almost half of individuals with ARC have had previous health care touchpoints for alcohol-associated diagnoses.^4^ Each of these represents a reachable moment, where clinicians have an opportunity to initiate effective treatment including MAUD, irrespective of whether the individual came in expressly asking for AUD treatment.

## MAUD IS UNDERUTILIZED

The low rates of treatment in this study are similar to those of others demonstrating underutilization of MAUD with only 4%–12% of individuals with AUD treated with pharmacotherapy.^5^ Clinicians miss opportunities to address alcohol use even when patients bring it up. For example, a study of primary care physicians found that patients share information about their alcohol use frequently, and yet, physicians often do not explore that information when shared, and when they do advice is vague compared with how physicians respond to tobacco use.^6^ Patients also share that they would feel comfortable discussing alcohol use with a physician they trust. For example, in a qualitative study on the topic a patient shared, “I’m very comfortable with my provider. That’s why I told her about what was going on. And so [in the appointment] I’m starting to open up to her and tell her the specifics of [my drinking] and … she listened, and was very compassionate, as always. [She] has been my care provider for the past 3 years, so … I’m very comfortable with talking to her about this subject”^7^. Although hepatologists frequently care for individuals with alcohol-associated liver disease, discomfort and lack of uptake around offering MAUD remain a challenge. A previous study looking at MAUD treatment among hepatologists found that, although 60% had treated a patient with MAUD, only 16.8% treated more than 20% of their active AUD population with MAUD.^8^

## HOW TO INCREASE MAUD INITIATION

In this study, prior inpatient AUD code and hospitalization were associated with higher odds of MAUD use. This emphasizes the importance of making MAUD standard practice for hospitalized patients with AUD. This is consistent with current consensus for other types of SUD, most notably opioid use disorder, where superior outcomes are observed when patients are started on medication during hospitalization or emergency department encounters and linked directly to ongoing care without interruption in pharmacotherapy.^9,10^ That model can and should be employed for AUD.

Increased MAUD initiation could be encouraged in 4 ways. First, MAUD initiation should be tracked as quality and contract performance measures. Second, addiction medicine education should be incorporated into all levels of undergraduate and graduate medical education to ensure that all physicians—hepatologists included—leave training with basic competency in treating AUD as they do for other prevalent health conditions. Third, clinical decision support should be built into electronic health records to make it easy for clinicians to do the right thing.

Finally, and most importantly, to increase the likelihood that hepatologists, and all physicians, will offer MAUD to a patient with AUD, we need to address the issue of AUD exceptionalism.^11^ The approach to AUD is no different than for any other chronic, treatable health condition physicians deal with regularly. We need to screen for it, diagnose it, start treatment expeditiously, and seek out expert care for more complex cases. Yet, study after study has demonstrated the many barriers that physicians and other healthcare clinicians identify when asked about treating AUD. The barriers cited for AUD treatment mirror those for other types of SUD and include worries about administrative burden, care interruptions, multimorbidity, and limited time to deliver direct care to patients.^11^ However, those barriers exist for all medical conditions that we treat, are not unique to AUD, and are rarely cited as a reason to not manage hypertension or HIV or cirrhosis.

The major challenge that we need to overcome is the ideology that we have all been exposed to as members of a society that is stigmatized toward SUD. As physicians, we have all been exposed to these biases, including the idea that people with AUD are to blame for their medical complications. Therefore, even when we start to talk about AUD as a health condition, we continue to treat people living with it as if it is an issue of bad behavior. One only has to look as far as our differential approaches to NAFLD compared with ALD to see the impact of these societal stereotypes. I have lost count of how many individuals I have personally seen declined for transplant and die from ALD in the hospital, while I have yet to see the same for someone with NAFLD.

This study adds to the growing body of evidence that, while ALD is a brutal public health and medical challenge to tackle, we have effective, lifesaving tools to utilize. MAUD has the potential to save lives. In the year 2023, offering it in all care settings should be the standard of care.
